# Acknowledging the Relevance of Elephant Sensory Perception to Human–Elephant Conflict Mitigation

**DOI:** 10.3390/ani12081018

**Published:** 2022-04-14

**Authors:** Robbie Ball, Sarah L. Jacobson, Matthew S. Rudolph, Miranda Trapani, Joshua M. Plotnik

**Affiliations:** 1 Department of Psychology, Hunter College, City University of New York, 695 Park Avenue, New York, NY 10065, USA; rball@gradcenter.cuny.edu (R.B.); sjacobson@gradcenter.cuny.edu (S.L.J.); mrudolph@gradcenter.cuny.edu (M.S.R.); mtrapani@gradcenter.cuny.edu (M.T.); 2 Department of Psychology, The Graduate Center, City University of New York, 365 Fifth Avenue, New York, NY 10016, USA

**Keywords:** elephants, sensory perception, human–elephant conflict, olfaction, audition, conservation

## Abstract

**Simple Summary:**

Elephants have a unique sensory perspective of the world, using their complex olfactory and auditory systems to make foraging and social decisions. All three species of elephants are endangered and inhabit environments, which are being affected rapidly by human development. Anthropogenic disturbances can have significant effects on elephants’ abilities to perceive sensory information and communicate with one another, potentially further endangering their survival. Conflicts over high-quality resources also arise from the overlapping habitation of humans and elephants. While many different methods have been employed to reduce this conflict, we propose that elephants’ unique olfactory and acoustic sensory strengths be considered in future mitigation strategies to achieve coexistence.

**Abstract:**

Elephants are well known for their socio-cognitive abilities and capacity for multi-modal sensory perception and communication. Their highly developed olfactory and acoustic senses provide them with a unique non-visual perspective of their physical and social worlds. The use of these complex sensory signals is important not only for communication between conspecifics, but also for decisions about foraging and navigation. These decisions have grown increasingly risky given the exponential increase in unpredictable anthropogenic change in elephants’ natural habitats. Risk taking often develops from the overlap of human and elephant habitat in Asian and African range countries, where elephants forage for food in human habitat and crop fields, leading to conflict over high-quality resources. To mitigate this conflict, a better understanding of the elephants’ sensory world and its impact on their decision-making process should be considered seriously in the development of long-term strategies for promoting coexistence between humans and elephants. In this review, we explore the elephants’ sensory systems for audition and olfaction, their multi-modal capacities for communication, and the anthropogenic changes that are affecting their behavior, as well as the need for greater consideration of elephant behavior in elephant conservation efforts.

## 1. Introduction

The three extant species of elephant—the Asian elephant, *Elephas maximus,* the African savanna elephant, *Loxodonta africana,* and the African forest elephant, *L. cyclotis*—represent some of the largest remaining megafauna in the modern age. Their sheer size (they are the largest living land mammals), their unique physical characteristics, their complex cognition, and their capacity for multi-modal sensory communication make them a fascinating subject of both public interest and academic research. Humans and non-human primates share a number of important behavioral and cognitive traits with elephants. For example, group size in several primate species and elephants changes dynamically over time (i.e., they live in fission–fusion social structures [1,2,3]). Both (particularly apes and elephants) have demonstrated a capacity for flexibility in cooperative interactions (e.g., [4,5,6,7,8]) and reassuring conspecifics in distress [9,10]. However, primates are primarily visual animals [11], while elephants prioritize olfactory and acoustic information [12,13,14]. These differences in sensory perspectives make it challenging to draw comparisons about animal intelligence and to study it directly, as research on these different species may require markedly different experimental approaches.

A greater understanding of elephant life histories and basic elephant anatomy, physiology, and behavior across all three species can have important implications for the design of basic and applied behavioral research studies. Asian elephants can weigh upward of 4000 kg [15] and typically spend over seventeen hours a day in the wild consuming over 150 kg of fresh, wet weight vegetation [16]. African savanna elephants can weigh more than 5000 kg [17] and spend eighteen hours a day consuming more than 250 kg of fresh vegetation [18]. Their size, as well as their substantial food consumption, mean they have a considerable impact on their natural environments. As a group of three keystone species, elephants also influence many other native species. Their tusks, for example, expose water underground during dry seasons, allowing other animals to hydrate. Their considerable appetite for vegetation clears plains on the savanna and trees in the forest, providing a clear area for predators to find prey [19]. They also disperse seeds across their ecosystems [20,21,22]. In this way, elephants contribute significantly to their native habitats and the species with which they share them.

Today, elephant habitats are heavily threatened by anthropogenic development. Rapid expansion of logging enterprises, agricultural and industrial development, and resource harvesting have all resulted in elephants being forced to use only a fraction of their potential habitats [23,24,25]. In Africa, 62% of the continent is potential elephant habitat, but elephants occupy only 17% of it [26]. In Asia, only 51% of the elephants’ range has large, unfragmented land suitable to support elephant populations [27]. The impact of humans on elephant habitat can have unintended behavioral implications as well. Noise and chemical pollution could cause auditory and olfactory disruption, making it difficult for elephants to perceive communication from conspecifics and natural environmental threats. Although these anthropogenic consequences have not been thoroughly explored in elephants, there is a strong theoretical basis for this, as well as evidence in other species [28,29,30]. Additionally, wild elephants from all three species are attracted to nearby agricultural fields, likely through their use of olfactory cues from the crops, resulting in clashes between the farmers that need the crops to make a living and the elephants that are feeding on the food for survival [31,32,33,34]. This leads to negative interactions between the farmers and elephants, as well as a conflict between different human interest groups that struggle to mitigate the situation.

As we discuss later in this review, there are many different mitigation strategies employed by farmers to try to prevent elephants from crop raiding, but the primary objective in many of these situations is to physically prevent interspecific interactions rather than to address the root causes of the conflict. While mitigation strategies may succeed or fail, in part due to the sensory information they manipulate, more attention is needed to the design of strategies that are intentionally ecologically salient. In fact, to date, there have been few attempts to prioritize the integration of knowledge about elephant behavior and sensory perception into conflict mitigation strategy development [34] (although see Ref. [35]), often complicating an already unstable coexistence between humans and elephants. The mutual behavioral flexibility of humans and elephants (i.e., their capacity to adapt to environmental variation) as they navigate an ever shrinking, shared natural habitat makes it even more difficult to develop long-term solutions to the conflict that provide for the needs of both parties. One challenge is to try to account for the elephants’ sensory perspective in their risk-taking behavior. How do elephants make decisions about raiding crop fields or interacting with humans, and how are these decisions influenced by particular types of sensory information?

### This Paper’s Purpose

This paper is not intended as a comprehensive review of what we know about elephant sensory perception (for that, see Ref. [14]). Instead, using general terminology intended for both a public and a scientific audience with open access to this paper, we present our perspective on encouraging active consideration of animal behavior and sensory perception in human–wildlife conflict mitigation and conservation efforts. Here, with a focus on elephants, we briefly highlight what we know about elephant olfactory and acoustic perception and then outline how such knowledge can better inform the human–elephant conflict in Asia and Africa. Acknowledging the elephants’ perspective in the conflict is crucially important if we are to have any chance of saving them from extinction.

We urge caution when generalizing the behavior and the underlying mechanisms of sensory perception to all three extant elephant species, and thus, in this paper, we (a) discuss audition and olfaction as important modalities for all elephants if there is common behavioral evidence, but (b) we distinguish between the study species when referring to specific research. We do generalize about the importance of olfaction and audition to elephants’ perception of the world and thus discuss the taxa at large when discussing the applications of behavior and sensory perception to human–wildlife conflict mitigation. The amount of research on the three species varies widely (with little data from African forest elephants to date). We recognize that future research may determine that any assumptions about similarities between the three species may need to be reconsidered.

## 2. Sensory Systems and Communication: Some Considerations

Elephants rely on olfaction as one of their primary sensory modalities [12,14,36,37,38]. They use olfactory information both to gather information about conspecifics [39] as well as to forage (e.g., [40]) with considerable research to date on the complexity of both functions. Male elephants experience musth, a period of heightened testosterone, aggression, and sexual activity [41,42] where they give off a distinct odor recognizable by both nearby males and sexually receptive females [43,44]. Elephants have an olfactory capacity for distinguishing between quantities of food (Asian elephants: [45]) and quality of food (African savanna elephants: [38,46]). Their auditory system is also complex, allowing for a broad repertoire of vocalizations between conspecifics both within and below the primate hearing range [47,48].

In the following sections, we will briefly review the olfactory and auditory abilities in elephants with detail about perception in both individual and social contexts. It is important to note that tactile (e.g., [49,50]) and visual (e.g., [51,52,53,54,55]) information is also important for elephants when navigating their physical and social worlds, and it is relevant for considering the roles of different senses when elephants engage with a variety of human–elephant conflict mitigation strategies (e.g., touch is likely important in conditioning for aversive stimuli, such as electric fences [50], and vision is important for the use of light-based deterrents [56]). In this paper, however, we focus only on the elephants’ olfactory and auditory senses (the most widely studied modalities in elephants) as examples for readers to understand the importance of considering the ‘elephant perspective’ in conflict mitigation.

The cerebellum, which may play an important role in sensory processing [57], comprises on average 18.6% of an elephant’s brain mass, compared to 10.3% in humans [58], suggesting a potentially unique integration of multiple sensory modalities. The perception of multi-modal cues (covering multiple senses) may provide elephants with complementary or more detailed information about their physical and social worlds. Remarkably, we still know relatively little about the interaction between sensory perception and cognition in elephants, particularly as it pertains to the elephants’ capacity to adapt to rapid environmental change. Because of the importance of olfaction to elephants in both physical and social cognition, we will start with what we know about the elephants’ sense of smell.

### 2.1. Olfaction

Elephant brains, containing an estimated 257 billion neurons [59], have the largest cerebral cortex of any terrestrial mammal, with a volume three times greater than that of humans [60] and an encephalization quotient (a brain size measure that accounts for body size) between 1.3 and 2.3, compared to 7.5 for humans and 2.5 for chimpanzees [61]. A significant portion of the cortex volume is dedicated to olfaction in elephants [58]. A high diversity of olfactory-related genes found in the African savanna elephant genome also suggests an overwhelming emphasis on smell as a predominant sensory modality [37]. Asian elephants have demonstrated a capacity for making precise discriminations between novel scents synthesized with structural similarities [62], and African savanna elephants can distinguish between familiar scents from different plant species [46]. Elephants also possess a vomeronasal organ, a separate chemoreceptive organ found in some olfactory systems that is used for specialized recognition of non-volatile chemicals [63]. This organ can be used to detect non-volatile components of odors that are transmitted through urine or the temporal gland, which may provide conspecifics with information about an elephant’s dominance or emotional state [43].

Recent experimental studies have shown that elephants use olfactory information to identify and gather information about food sources. For example, Asian elephants can differentiate quantities using smell alone [45], an olfactory ability that is comparable to visual discrimination made by other mammals, such as orangutans [64], sea lions [65], and bottlenose dolphins [66]. Additionally, African savanna elephants can use olfactory cues to choose their preferred food even when masked with odors of non-preferred food [67]. To further highlight how these discrimination skills may be olfactory specific, Asian elephants were able to make clear choices about the location of food in object choice tasks when olfactory, but not visual or acoustic information, was available [40]. Similarly, African savanna elephants can make preferential foraging decisions based solely on olfactory cues from distances seven meters away [46], suggesting the elephants’ sense of smell is important in their decision-making process in a variety of contexts.

Elephants’ olfactory perception contributes both to their interactions with the natural environment, as well as to their relationships with others. Elephants’ numerous relationships require them to keep track of many individuals and to communicate with one another over long distances and time scales [68,69,70]. For example, Bates et al. [39] showed that African savanna elephants are capable of keeping track of out-of-sight family members by processing olfactory information left behind (urine), and Rasmussen et al. [43] have shown that male African savanna elephant bulls likely avoid conflict with other males based on information gathered from musth secretions. The establishment of dominance relationships between bulls may lead to costly aggression, which can sometimes prove deadly [42,71,72]. To circumvent this, olfactory cues associated with musth (such as secreted temporal gland fluids and dribbled urine providing information about testosterone levels [42,73,74]) may act as a signal to establish dominance relationships and deter interactions between potential rivals [43,74]).

Reproductive signaling, which involves the use of secretions in order to communicate information about estrus or breeding receptiveness (reviewed in Ref. [75]), is also an important form of olfactory communication between elephants. Asian elephant females’ urine during periods of estrus, for example, provides information for passing bulls about sexual receptivity [36]. Similarly, olfactory information emitted by males (e.g., the strong odor from musth bulls) alerts nearby receptive females of their presence. The vomeronasal organ is particularly important for communication between elephants in regard to sexual behavior [76] and has a role in the discrimination of musth chemo-sensory information integral to sexual selection in elephants [77,78,79].

### 2.2. Audition

The elephants’ specialized ears provide an acoustic complement to their sense of smell, allowing elephants to gather an extraordinary amount of multi-modal sensory information [14]. The auditory system is particularly sensitive to low-range sound frequencies. Elephant ears have a flexible range of motion that aids in sound localization, large pinnae that act to funnel sound, and enlarged middle ear structures that better aid in the detection of a range of frequencies that enable an elephant to gather substantial acoustic information in diverse landscapes [80,81]. Elephants perceive auditory information from both conspecifics and allospecifics, with cues from the latter allowing for the perception of potential threats. Although adult elephants typically have no natural predators, big, wild cats from the *Panthera* genus, such as tigers (*Panthera tigris)*, are known to opportunistically hunt Asian elephant calves [82,83]. While not a predator, bees may convey the potential threat of being stung, which appears to be a strong aversive stimulus for elephants resulting in their moving away from the sound of beehives [84,85,86].

Both acoustic and olfactory information from potential predators can impact elephant behavior, potentially deterring elephants from traveling to vulnerable areas, such as water holes, or encouraging them to be more vigilant around young calves within their family groups [87,88]. As the threat of humans to elephant habitat increases, African savanna elephants have learned to avoid human-generated seismic information [30] and to differentiate between threatening and non-threatening human ethnic groups using multi-modal sensory signals (visual and olfactory [12]; auditory [13]). Olfactory and acoustic information likely complement one another in guiding an elephant’s decision making, particularly in reaction to potential threats, but also in helping to regulate relationships between both unknown and closely bonded individuals.

Acoustic signals are particularly important for elephants in terms of social communication within and between family groups. Elephants produce different kinds of vocalizations, ranging from higher frequency ‘chirps’ or ‘squeaks’ (only observed in Asian elephants [10,80,89,90]) to rumbles and other lower frequency calls in the infrasonic range (Asian [47], African savanna and Asian [91], African forest [92]). Elephants are less sensitive to higher frequency sounds and considerably more sensitive to lower frequencies [93]. These low frequency vocalizations or rumbles can travel along the surface of the earth as seismic waves and may be received through elephants’ foot pads [94]. Research with African savanna elephants has demonstrated that infrasonic vocalizations allow for communication of signals over long distances [95], although information may be lost as distance increases [96]. These infrasonic vocalizations do not travel as far for African forest elephants [97] so may not be as useful for long-distance communication in forested environments.

Vocalizations are used for a wide variety of social functions. Calls can be used to differentiate familiar from unfamiliar conspecifics, which is useful for potential mating success, as has been observed in African savanna elephants [98]. Reproductive coordination can be achieved between ovulating females and unrelated males using vocalizations over short and long distances [68,99]. Female African savanna elephants in mid-estrus use loud, low frequency calls to attract multiple males to their location, who then may compete for breeding access [42,100]. Male African savanna elephants use similar ‘musth rumbles,’ which are low-frequency calls that advertise sexual state [42,101,102]. Elephants also respond to conspecific vocalizations either to find associated kin for safety or to avoid unknown adversaries [90,103,104]. Thus, acoustic information can be beneficial for both familiar and unfamiliar interactions.

## 3. Impact of Anthropogenic Change

### 3.1. Overview

Anthropogenic development, particularly human encroachment on wild animal habitat, leads to significant changes in natural ecosystems, to which animals, including elephants [23], struggle to adapt [105]. Deforestation and poaching of central Africa’s forest elephants, for example, disrupt their fission–fusion associations and ability to maintain complex relationships with other individuals [24]. African savanna elephant groups that lose matriarchs or other experienced, older elephants struggle with social integration and access to resources [106,107], as well as to distinguish predatory threats [108]. These changes can directly impact elephant survival, but there may also be more indirect impacts of anthropogenic disturbance on elephant fitness because of their unique sensory systems. Due to the predominant importance of olfaction and audition to elephant behavior, we discuss potential anthropogenic effects on these sensory systems here.

### 3.2. Impact on Olfactory Environment

As outlined above, elephants use their olfactory systems when making foraging decisions [46], as well as to provide cues to each other about identity, body state, emotion, and dominance [36,39,43,74]. Anthropogenic influences, such as pollution and industrialization, can result in olfactory distractions with new scents that negatively impact many species’ capacities for olfactory-based discriminations and navigation [28]. Although this influence has not been studied directly in elephants, it is likely that they are also affected. In fact, to our knowledge, the disruption of such information through chemosensation has not been studied in detail in any terrestrial mammals. However, it is likely that the perception of chemical information could be disturbed by pollutants in similar ways across species (e.g., pesticides impairing pheromone communication in newts [109]; pollutants affecting olfactory navigation in moths [110]). While studying chemosensory information and disruption in forest or savanna environments would be difficult, this is an area of research that warrants further study.

**Figure 1:**
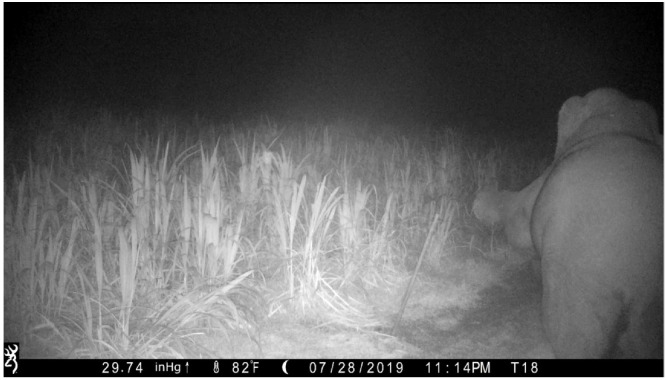
A still from a camera trap video taken along the periphery of a crop field in Thailand. The bull elephant is using his foot to snap the wire of an electric fence in order to enter a sugar cane field. The elephant regularly forages in this field at night, suggesting he uses olfactory information to locate the sugar cane. Video recorded by the authors.

Deforestation leads to a decline in availability of food and water resources [25,26], while new agricultural developments continue to expand in proximity to wild elephant populations and their habitat [111]. Farms in close proximity to wild elephants may contain crops that are olfactorily perceptible to elephants, even from a distance [32]. With the added challenge of a declining abundance of natural resources, particularly potent crops become high-value targets for elephant crop raiding. Pineapples, bananas, sugarcane, papayas, coffee, and spices, for instance, represent some of the crops grown close to wild elephant habitat in Asia [32,111] and are particularly attractive to Asian elephants. These crops have high nutritional value, greater water content, and weaker chemical defenses than wild browse plants [32,112,113,114], and because they are concentrated in a relatively small area, elephants may be willing to take on higher risk to consume them [32]. African elephants can distinguish higher levels of sugar content in crops from scent alone [38], suggesting the smell of crops is likely what first attracts them to the crop fields (Figure 1), even if mechanisms such as social learning often lead to other individuals or groups following suit once highly attractive resources have been identified [115]. Olfactory cues could also be responsible for attracting Asian elephants to garbage dumps, where consumption of foods with high nutritional content can lead to improved body condition [116]; unfortunately, there may also be a risk of health issues from accidental consumption of non-food items in these dumps (similar to the negative impact of such consumption on nutrient absorption and endocrinology in marine mammals [117]).

The elephants’ attraction to crop fields and human habitat increases the frequency of negative interactions between the species and influences elephant social behavior. Male elephants are the most common crop raiders, either entering crop fields alone or in small bachelor herds of several individuals [33,118]. Consuming these easily accessible and highly nutritious crops may also improve the elephants’ reproductive success by enabling bulls to stay in musth longer and prolong the mating period [33,118]. This is an incentivized mechanism, wherein the presence of crops encourages reproduction by those crop-raiding bulls, making the behavior ecologically favorable despite negative human interactions.

### 3.3. Impact on Auditory Environment

Whereas olfactory stimuli from crops attract elephants into anthropogenic landscapes, the sounds of human activity may repel elephants away from them [30]. This may be one reason that most crop raids actually occur at night [32], when humans are likely asleep, and noise is minimal. With rapid construction and development in wildlife habitats, increased noise pollution undoubtedly has an impact on elephant behavior. For example, Asian elephants are able to discriminate different sources of seismic auditory cues and show avoidance behaviors in response to human-generated sounds [30].

Anthropogenic activities may act as ‘noise’ to signals [119], such as those communicated between elephants. As human development expands and elephant habitats diminish [26], extraneous human auditory noise could interfere with elephants’ vocal signals, as has been demonstrated for other species (reviewed by Ref. [29]). We know that elephants are sensitive to auditory cues from their environment [120], including from conspecifics, predators, and humans. Just as any environmental noise makes signaling less effective [119], elephants’ long-range communication could be masked by any human activity that produces low-frequency noise, thereby potentially altering the elephant’s ability to recognize or communicate with individuals, maintain hierarchies, or make mating decisions. This type of auditory masking has previously been observed to disrupt communication in birds [121] and ground squirrels [122], but low-frequency noise from ground vehicles or planes—which produce sounds that may resemble rumbles or are difficult for elephants to localize—may theoretically cause the most significant disruption for elephants. Overall, human development disturbs the auditory environment for wild elephants with the introduction of novel sounds, which may induce fear and stress [34,123,124]. These sounds may also cloud the ability to process signals intended for others [30,116,120], potentially posing a risk to elephants’ ability to transfer information between individuals.

## 4. Human–Elephant Conflict and Mitigation

### 4.1. Overview

The IUCN’s Human-Wildlife Conflict Task Force describes human–wildlife conflict (HWC) as ‘struggles that emerge when the presence or behavior of wildlife poses actual or perceived, direct and recurring threat to human interests or needs, leading to disagreements between groups of people and negative impacts on people and/or wildlife’ [125]. While this definition emphasizes the fact that HWC is most often expressed as interactions between humans over wildlife, animals can sometimes be parties to the conflict as well, particularly when the target species is cognitively complex and adapts quickly to human behavior.

In the majority of incidents, elephants are not active participants in conflict, as their behavior is driven by a desire to seek high-quality resources rather than a choice to engage in negative interactions with humans. In addition, there is variation in whether individual elephants choose to access resources in human-dominated landscapes (e.g., [26,34]), which is likely dependent upon factors such as energy expense [126], hormonal changes [127], and possibly personality traits [34]. Unfortunately, unpredictable and rapidly expanding human encroachment on wild habitat often makes it more difficult for elephants to avoid the conflict entirely.

Risk, or even the perception of risk, can shape a mammal’s movement patterns [128]. Interactions between humans and elephants are inherently risky [129], but the availability of high-quality food in easily accessible habitat (crop fields) often has a significant impact on elephant foraging strategies and movement patterns [130]. For an elephant to assess risk adequately, they need to be aware of the kinds of risks that exist. Several predator traits, such as speed and size, are important for risk assessment in many species of prey [131], but the cues that might influence how elephants determine their vulnerability to human threats have not yet been investigated.

One way of conceptualizing human–elephant conflict (HEC) mitigation—physical measures employed to prevent elephants from entering human-dominated habitat [56,132]—is to consider how best to provide elephants with options that affect their decision-making processes in ways that prevent negative interactions with humans. This consideration should take existing knowledge from the animal behavior literature into account, as it is particularly relevant for understanding how animals, including elephants, make decisions.

As for any animal, the elephant’s decision-making process can be complex [133]. We must consider the physical and social environments, including those shaped by anthropogenic changes, and how they impact the sensory, cognitive, and behavioral factors involved in an elephant’s decision to take risks in search of desirable crops fields. The anthropogenic environment may influence the ‘animal’s decision-making’ process or ADM [134] in a variety of ways. For example, ‘comparative valuation’ explains how animals choose between two or more options of different value that are simultaneously available [133,135]. An elephant, for instance, may perceive both a human crop field and a wild source of vegetation at the same time, preferring the higher quality (or more salient) crop field. Choosing the latter may also be explained by signal detection theory, which focuses on how animals may perceive sensory cues when these cues are obscured by background noise [136], as elephants’ decisions about entering human habitat may be influenced by the presence of other aversive cues (e.g., other elephants, unpalatable crops, or people) that may influence the salience of the crop odors. The outcome of an individual’s decision to crop raid (i.e., whether it was successful) will also likely influence future decisions about whether to repeat this behavior. If an elephant raids a crop field and consumes a desirable yield with minimal perceived threat, then ‘reward-guided learning’ may occur [137], reinforcing the elephant’s ‘good’ decision to crop raid. While human stakeholders may not often consider the theoretical aspects of the animal decision-making process in detail, elephant researchers should emphasize the importance of behavior in understanding the elephant perspective in HEC.

**Figure 2:**
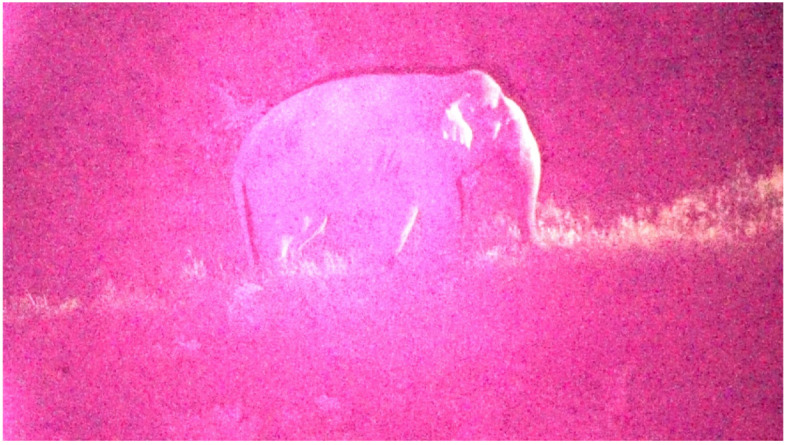
A video still of a bull elephant in a crop field in Kanchanaburi, Thailand. The pink hue is from an infrared camera; the white spotlight is attached to a farmer’s truck and is intended as a deterrent. The sound from the truck’s engine and shouts from the farmers also act as significant aversive stimuli. Video recorded by the authors.

To be successful in the long term, HEC mitigation techniques must help convey risk in a sensory modality that is both relevant to and interpretable by the elephant. While visual deterrents, for example, are often used by farmers and can be successful (e.g., flash- or spot-lights [56]; Figure 2), we want to highlight how the two predominant sensory modalities for elephants can be integrated into HEC mitigation and how their use could be expanded for future efforts aimed at promoting coexistence between species.

### 4.2. Olfaction-Dependent Mitigation

Current sensory-based mitigation techniques often promote the use of deterrents or repellents to prevent elephants from coming into close contact with human development [75,132,138,139,140]. One technique with evidence of success both in Asia and Africa is the use of burning chilies (*Capsicum* spp. [132,139,140,141]). These chilies emit a strong and unpleasant smell via their activated capsaicin compound. Capsaicin is an unattractive and repelling scent to elephants [142]. There are differing methods of applying capsaicin; grease from chilis, tobacco, and engine oil is spread across rope fences [132] or burned in briquettes of chilies and elephant dung around fences [139]. The smoke produced by igniting capsaicin causes a burning sensation in the trunk and stimulates the cranial nerve [142,143], likely deterring the elephant from further foraging. As elephants have complex olfactory systems that help them discriminate between desirable and undesirable odors [12,58,67], capsaicin (representative of the latter) could be useful in diverting them from crop fields.

Unfortunately, however, this chili-based mitigation technique has shown variable success across landscapes. Some locations, such as in Kenya [132] and Zimbabwe [144], have seen remarkable consistency in the efficacy of chili as a deterrent. Other locations have seen less success, such as in Botswana [140], where researchers found that the strategy is only effective as a repellent when chili briquettes are actively smoldering [140]. Nonetheless, the technique remains promising because (a) it has the potential to reduce the chance of conflict through non-violent means, and (b) the resources needed to implement it are readily accessible for many local communities impacted by elephants [132].

The chili-based techniques are typically installed from within the cropland [140] or around the farm’s perimeter [132,139]. When the elephant is at the edge of a crop field, they can likely detect the desirable food items contained within [46], even if the crops are surrounded by aversive stimuli, such as chilies. The elephant is then caught between the aversiveness of the deterrent and a desire for the reward (the high-quality crop) that lies beyond it. Thus, future mitigation strategies, including those that have found wide success, such as chili-fencing, should consider this balance in their design. When deterrents are installed close to the high-quality food, the olfactory influence of the ‘reward’ may be strong enough to encourage the elephants to circumvent them. However, if the deterrents were installed farther away, perhaps before the palatable crops could be detected or at least where the strength of the olfactory cues emanating from them was reduced, the effect of the aversive stimulus on the elephant’s decision may be stronger.

Considering the importance of olfactory cues for social signaling in elephants, another promising direction for impacting elephant decision making may be to manipulate the presentation of social chemicals to deter elephants from particular areas (see Ref. [75] for more discussion). For example, because younger African elephant males have been observed avoiding the musth signals of older, more dominant bulls [43], future research could investigate the placement of musth cues in strategic locations to deter elephants from entering crop fields where they may now believe a potential rival is foraging. Predator odors may also have a similar repelling effect for family groups with calves that are at risk of predation [88]. These potential mitigation strategies take the sensory perspective of the elephant into account and recognize the animal’s need to balance risk and fitness benefits as it navigates complex, volatile landscapes. Recognizing human–elephant conflict as a ‘two-species problem’ is crucial for the development of future mitigation strategies that aim for longer, more sustainable solutions.

### 4.3. Audition-Dependent Mitigation

The use of auditory deterrents has been traditionally rooted in local, community-based knowledge that is shared as best practice when successful [145]. These techniques range from setting off firecrackers to hitting metal objects together [145,146]. Recently, the use of modern technology has also been employed to redirect elephants, such as with buzzing unmanned aerial vehicles [147]. As we have discussed, elephant vocal communication and acoustic signal interpretation facilitates individual recognition [98], as well as perception of potential predatory and environmental threats, such as bees [35,84,85] and tigers [83]. Rather than using unnatural sounds, such as gunshots [24], naturally occurring, socially relevant sounds, such as calls from a conspecific, may be more salient to elephants and produce a more instinctive or natural aversive response [148]. The message communicated to elephants should be ‘in their language’; in other words, strategies that are ecologically relevant to elephants—such as calls or smells from conspecifics or allospecifics—may have greater potential for long-term success, although further research is needed to determine how the effects of these stimuli vary within and between elephant populations.

Because predator playbacks (i.e., playing aversive sounds of predators experimentally to see how potential prey respond) can result in the exhibition of defensive behaviors by African savanna elephant family groups (e.g., [149]), it is likely these signals could also act as an effective HEC mitigation strategy. For example, Thuppil and Coss [83] found that playing predator vocalizations—in this case, tigers and leopards—was effective at reducing crop raiding in India even for male Asian elephants, although there were differing levels of habituation over time based on the particular predator. It would be beneficial to further investigate the effectiveness of predator playbacks in changing the movement patterns of elephants in and around crop fields, as well as to determine whether habituation to predator sounds could be avoided by varying how and when such sounds are played. Habituation is of particular concern, especially if the cues or schedule of playbacks are not varied, because it could desensitize the elephants to natural threat signals. This could diminish the elephants’ anti-predator responses to actual predator calls, increasing potential danger. Researchers and wildlife managers should use these cues sparingly to avoid altering the elephants’ natural aversion to them in the wild.

The simulation of conspecific vocalizations could be both threatening or non-threatening, with the latter more likely to have long-lasting, positive effects on elephant behavior. While males may typically be attracted to female-led family groups for mating opportunities when there are individuals in estrus [1,68,150,151], these groups may not tolerate bulls who linger too long nearby [44]. Therefore, a bull could, at certain times, respond more aversively to a family group of elephants than a lone female, possibly resulting in his avoidance of group vocalization playbacks. Indeed, in a study by Wijayagunawardane and colleagues [148], the most effective sound used to deter Asian elephants away from human activities was a simulated elephant matriarchal group. Using a mitigation technique that relies upon the natural social behavior of elephants—i.e., the differing life histories of solitary males and female-led family groups [44,150,152]—may prove effective in deterring elephants from human settlements. On the other hand, it is also important to consider that the use of social signals could have unexpected effects on natural elephant social behavior. This too would need to be explored when studying elephant behavior in relation to conflict mitigation and its long-term effects. While the use of conspecific calls is a novel mitigation technique, it is also possible that elephants could habituate to the calls quickly, resulting in only short-term changes to their behavior. Future research could expand upon the findings of Wijayagunawardane and colleagues [148] to develop further integrations of natural elephant behavior and communication in HEC mitigation.

## 5. Conclusions

The success of HEC mitigation depends on a thorough and scientific integration of two primary components—how elephants send and receive sensory information and how ongoing anthropogenic activities impact their perception of it. The difficulty in trying to ‘solve’ HEC is in determining how to address and balance the needs of both humans and elephants. This requires an active collaboration between stakeholders, including governments, local communities, and scientists, where the latter’s role is to identify the behavioral and ecological impacts on both species. Most mitigation strategies aimed at preventing elephants from entering human-dominated habitats are human centric and only focus on deterring elephants rather than understanding how these strategies impact or account for their sensory perspectives. As we have learned more about the behavioral flexibility of elephants and how it enables them to adapt to rapid environmental change, it has become apparent that many mitigation techniques in place today will likely fail in the long term. Current approaches that rely on certain types of deterrents or aversive stimuli [140,145,147] could result in elephants becoming habituated or desensitized to signals that initially discouraged them from entering human habitat (e.g., [153]). Thus, moving forward, we need a new approach that considers the elephants’ decision-making process and risk assessment when navigating shared habitat [129]. Mitigation techniques may be most successful when they are not only able to provide salient signals of risk, but also when they consider the importance of providing adequate food, water, and other landscape-specific resources for the elephants. It will also be important to consider individual variation in elephant behavior and cognition [34], as it is possible that how elephants respond to mitigation strategies is dependent on differences in personality, experience, and life history as well. To help human societies flourish without harming elephants, we should prioritize an application of elephant behavior and ecology to HEC mitigation that includes consideration of their ears, trunks, and minds as part of the conversation.
